# Contributing factors for acute stress in healthcare workers caring for COVID-19 patients in Argentina, Chile, Colombia, and Ecuador

**DOI:** 10.1038/s41598-022-12626-2

**Published:** 2022-05-19

**Authors:** Jimmy Martin-Delgado, Rodrigo Poblete, Piedad Serpa, Aurora Mula, Irene Carrillo, Cesar Fernández, María Asunción Vicente Ripoll, Cecilia Loudet, Facundo Jorro, Ezequiel Garcia Elorrio, Mercedes Guilabert, José Joaquín Mira

**Affiliations:** 1 Atenea Research Group, Foundation for the Promotion of Health and Biomedical Research, Carretera Nacional 332, Hospital Universitario de Sant Joan d´Alacant, 03550 Sant Joan d´Alacant, Alicante, Spain; 2 Instituto de Investigación E Innovación en Salud Integral, Facultad de Ciencias Médicas, Universidad Católica de Santiago de Guayaquil, Guayaquil, Ecuador; 3 Pontifical Catholic University of Santiago de Chile, Región Metropolitana, Chile; 4 Universidad de Santander, Bucaramanga, Colombia; 5 Department of Health Psychology, Miguel Hernández University, Elche, Spain; 6 General José de San Martín de La Plata General Hospital, Buenos Aires, Argentina; 7 Institute for Clinical Effectiveness and Health Policy (IECS), Buenos Aires, Argentina; 8 Hospital General de Niños Pedro de Elizalde, Buenos Aires, Argentina; 9 Health District Alicante-Sant Joan, Alicante, Spain

**Keywords:** Health services, Occupational health

## Abstract

This study analyzed the frequency and intensity of acute stress among health professionals caring for COVID-19 patients in four Latin American Spanish-speaking countries during the outbreak. A cross-sectional study involved a non-probability sample of healthcare professionals in four Latin American countries. Participants from each country were invited using a platform and mobile application designed for this study. Hospital and primary care workers from different services caring for COVID-19 patients were included. The EASE Scale (SARS-CoV-2 Emotional Overload Scale, in Spanish named Escala Auto-aplicada de Sobrecarga Emocional) was a previously validated measure of acute stress. EASE scores were described overall by age, sex, work area, and experience of being ill with COVID-19. Using the Mann–Whitney U test, the EASE scores were compared according to the most critical moments of the pandemic. Univariate and multivariate analysis was performed to investigate associations between these factors and the outcome ‘acute stress’. Finally, the Kruskal–Wallis was used to compare EASE scores and the experience of being ill. A total of 1372 professionals responded to all the items in the EASE scale: 375 (27.3%) Argentines, 365 (26.6%) Colombians, 345 (25.1%) Chileans, 209 (15.2%) Ecuadorians, and 78 (5.7%) from other countries. 27% of providers suffered middle-higher acute stress due to the outbreak. Worse results were observed in moments of peak incidence of cases (14.3 ± 5.3 vs. 6.9 ± 1.7, *p* < 0.05). Higher scores were found in professionals in COVID-19 critical care (13 ± 1.2) than those in non-COVID-19 areas (10.7 ± 1.9) (*p* = 0.03). Distress was higher among professionals who were COVID-19 patients (11.7 ± 1) or had doubts about their potential infection (12 ± 1.2) compared to those not infected (9.5 ± 0.7) (*p* = 0.001). Around one-third of the professionals experienced acute stress, increasing in intensity as the incidence of COVID-19 increased and as they became infected or in doubt whether they were infected. EASE scale could be a valuable asset for monitoring acute stress levels among health professionals in Latin America.

*ClinicalTrials*: NCT04486404.

## Introduction

The front line of care professionals for COVID-19 patients has to experience extreme emotional overload that causes acute stress reactions, compassion fatigue, and other affective pathologies and adaptative responses^1–4^. This situation may negatively affect the quality of health care received by patients. Furthermore, it may impact physicians’ abandonment rate or transfer to less compromised destinations, unbalancing health care systems.

Uncertainty about the most appropriate approach to the COVID-19 pandemic, the scarcity of resources (mechanical ventilators and medicines), and the breakdown of the supply chain limiting the availability of personal protective equipment during the onset of the outbreak^5,6^, have affected labor morale^7,8^ and have been associated to the emergence of adaptive disorders, post-traumatic stress^9^ moral injury^10^ and burnout^11^.

To this impact on mental health, the number of healthcare professionals who have been infected and seen their lives in danger must be added. Also, those in isolation because of close contact with confirmed cases of COVID-19 had experienced the fear of the disease, especially during the first outbreak when the lack of equipment was most significant^4,11^.

For these reasons, healthcare professionals have been considered second victims of SARS-CoV-2^12–14^ and are offered psychological support to cope with this experience^15^. However, many of these professionals have been reluctant to seek this initial psychological help, so other alternatives have been developed^16^. In this context, the self-applied SARS-CoV-2 Emotional Overload Scale (EASE) was designed and validated in Spain to help professionals become aware of the level of stress they were experiencing in the care of COVID-19 patients^17^.

Some studies have suggested that the impact of the COVID-19 pandemic on the mental health of health professionals may be different depending on the country and its resources, the care activity carried out, or the time of the evaluation, among other factors^18,19^. Almost all studies have been carried out in China and developed Western countries such as Germany, Italy, Spain, or the United States of America^5,20–22^. In Latin America, the number of studies is limited, and they have a more significant shortage of personal protection equipment and diagnostic means^23^.

This study used the EASE scale to analyze the frequency and intensity of acute stress among healthcare professionals in Latin America. Secondary objectives were to test the hypotheses that acute stress was related to (i) time points in the pandemic of the highest incidence of COVID-19, (ii) place of work/direct care of COVID-19 patients, and (iii) the experience of being ill during the pandemic.

## Methods

A cross-sectional study was conducted, involving healthcare professionals from four Latin American Spanish-speaking countries: Argentina, Colombia, Chile, and Ecuador. These four countries were selected because the COVID-19 pandemic has caused more than 94,884 deaths and a cumulative incidence of 3,120,585 cases reported as of November 13, 2020^24^.

The field study was conducted in Argentina, Colombia, and Chile between May 1 to September 30, 2020. And in Ecuador, it was conducted between April 8 to August 30, 2020. Digital informed consent was obtained from all participants before completing the questionnaire. Participation was voluntary, and they could withdraw at any moment just by closing the questionnaire. No personal identification data were included.

### Materials

The EASE scale^17^ has been previously developed and tested in a Spanish sample (primary care and hospital staff). EASE was specifically designed to assess the possible acute stress reactions experienced during the pandemic by COVID-19. It consists of a self-applied 10-item tool with four alternative Likert-type responses (it is not happening to me—I am like this all the time). The scale score can range from 0 to 30 points, and it is structured in two factors that explain 55% of the variance. Factor 1 refers to the emotional response and comprises six items (0–18 points), and factor 2 to the fears and anxiety experienced during the care of COVID-19 patients and adds up to 4 items (0–12). The interpretability of the score is prioritized into four categories, good emotional adjustment (0–9 points), emotional distress (10–14 points), high emotional distress (15–24 points), and acute stress (> 25 points). Considering that there could be differences in the Spanish spoken in each country, we first checked the readability of the questions and their relevance for each of the four countries. For this purpose, an analysis of the linguistic and socio-cultural equivalence of each of the items in the scale was conducted. Three professionals from Argentina, four from Colombia, eight from Ecuador, and two from Chile reviewed the expressions, terms used, and the context to which they alluded. Where appropriate, they proposed modifications, justifying them. The research team, by consensus, determined the changes in the wording of the items. Furthermore, the metric properties of the EASE scale were tested to ensure its reliability and validity. The internal consistency of EASE was calculated using Cronbach's Alpha (0.85) and McDonald's Omega (0.87). The construct validity of the scale was determined by confirmatory factor analysis that employed the following indices: comparative fit index (0.93), goodness of fit index (0.93), root mean square error of approximation (0.085), standardized root mean square residual (0.06), normed fit index (0.90). Scores higher than 0.90 for CFI, AGFI, and GFI and lower than 0.8 for RMSEA and SRMR suggested a good model fit.

### Procedure

Responses to the revised version of the EASE scale were collected through a link to an online platform designed for this study or by downloading an application (available for iOS and Android devices) that presented each of the items and made it easier to respond^16^. Up to 5 reminders were made in each of the countries involved. Once the required sample size was reached, the platform did not support more responses. The same IP address was not allowed to respond more than once to avoid duplication.

### Sampling

Stratified sampling was applied considering the country of residence. A convenience sampling was carried out. A total sample size of 1256 professionals involved in the care of COVID-19 patients was estimated for ± 3% accuracy, 95% confidence level, p = q = 50%, and 15% non-response ratio.

### Participants

Participants were recruited through institutional mailings, instant messaging applications, and discussion forums. Balanced participation of primary care and hospital professionals, public and private institutions, and the following healthcare workers, including doctors, nurses, and health technicians, was sought. Physicians in training were also invited. Other variables collected were the area of work (e.g., emergency room, intensive care unit), occupational exposure to COVID-19 patients (conducting interviews, caring for COVID-19 patients, performing higher-risk care such as patient intubation, airway care, nebulization, or others involving aerosolization), whether if they had been infected with COVID-19 or not.

### Statistical analysis

The World Health Organization database was used to obtain data on SARS-2-CoV incidence and lethality to guide comparisons in outcomes between countries. The following indicators were considered (i) total SARS-CoV-2 confirmed cases per million inhabitants, (ii) total SARS-CoV-2 deaths per million inhabitants, (iii) total SARS-CoV-2 deaths per thousand of SARS-CoV-2 confirmed cases. Using this information, a daily report was made for each participating country. Furthermore, rates were attenuated to graphic incidence curves for a more straightforward interpretation of data. As each participating country had different behaviors, the moment of highest pressure was chosen as the peak of incidence cases per million inhabitants. The period of the initial increase in incidence, peak incidence, and subsequent decrease of incidence was selected as the "most critical moment of the pandemic" as the highest pressure for assistance and daily deaths due to COVID-19 increased.

Variables of interest followed a Poisson distribution. Furthermore, variables were assessed for normality using the Kolmogorov–Smirnov test. Since the variables of interest do not comply with the normality assumption, an independence/homogeneity study was performed before using nonparametric tests. First, EASE scores were described overall, and by age, sex, work area (representing direct care of COVID-19 patients or not), and experience of being ill with COVID-19. Using the Mann–Whitney U test, the EASE scores were compared according to the most critical moments of the pandemic (peak incidence and subsequent decrease of incidence), considering the incidence and daily number of deaths of COVID-19 patients in each country. In this analysis, the data for Argentina was limited to the initial increase in incidence and peak incidence phases, considering the COVID-19 incidence data that at the date of this study showed an upward curve. Univariate analysis was performed to investigate associations between these factors and the outcome “acute stress”. Two multivariable logistic regression analyses were performed, the first to investigate whether the place of work/direct care of COVID-19 patients was associated with acute stress (this is the dependent or outcome variable), and the second to investigate whether the experience of being ill in the pandemic was associated with acute stress, adjusting for relevant confounding factors. The statistical significance was determined using multivariable logistic regression at 95% confidence intervals (*p*-value < 0.05 two-tails). Finally, using the Kruskal–Wallis test, EASE scores of those who reported having suffered COVID-19, who were not infected, and who had doubts about whether they were infected were compared. The coding and analysis of the data were carried out using IBM SPSS Statistics software, version 25.

### Ethics approval

The Research Committee of the San Juan University Hospital in Alicante (April 8, 2020) and the Scientific Ethics Committee from the Pontifical Catholic University of Chile (200630029) approved the study protocol in accordance with the Declaration of Helsinki.

## Results

A total of 1372 professionals responded to all the items in the EASE scale: 375 (27.3%) Argentines, 365 (26.6%) Colombians, 345 (25.1%) Chileans, and 287 (20.9%) Ecuadorians. The majority (1009, 73.5%) worked in hospitals. Only 638 professionals responded to the question of whether they had been infected (Table 1).

**Table 1:** Description of the sample.

	N	%
**Country**		
Argentina	375	27.3
Chile	345	25.1
Colombia	365	26.6
Ecuador	287	20.9
**Scope**		
Primary care	221	16.1
Private hospital	723	52.7
Public hospital	286	20.8
Others	142	10.3
**Sex**		
Men	365	26.6
Women	1007	73.4
**Profession**		
Specialist doctor	173	12.6
Medical doctor	532	38.8
Nurse	300	21.9
Nursing assistant	78	5.7
Others	289	21.1
**Work area**		
Emergency Room	187	13.6
Outpatient consultation	217	15.8
Specific hospitalization for COVID-19 patients	143	10.3
Non-COVID hospitalisation	92	6.7
COVID-19 Critical care (includes ICU or intermediate)	250	18.2
Home and/or ambulance service	49	3.6
Not just a single destination (hospitalization, consultation, residence)	434	31.7
**COVID-19 infection**		
No	363	26.5
Yes	149	10.9
Do not know	126	9.2
No reply	734	53.5
Age (mean, SD)	36.9	10.0
Years in the profession (mean, SD)	11.7	9.5
Total	1372	100

### EASE descriptive statistics

The average score on the scale was 10.6 points (SD 6.9, IC95% 10.2—11.0). Most professionals scored between 0 and 9 points, good emotional adjustment (671, 48.9%), 332 (24.2%) between 10 and 14 points, emotional distress, 318 (23.2%) between 15 and 24 points, medium–high emotional overload, and 51 (3.7%) 25 or more points, extreme acute stress.

Fear of infecting the family when returning home, not being able to disconnect once outside work, having lost calmness or ability to enjoy everyday things, or feeling that you have failed to people who need your help were the most common feelings. Supplemental Table 1 contains descriptive information on each of the EASE scale items.

### Incidence and lethality of SARS-2-CoV

**Figure 1:**
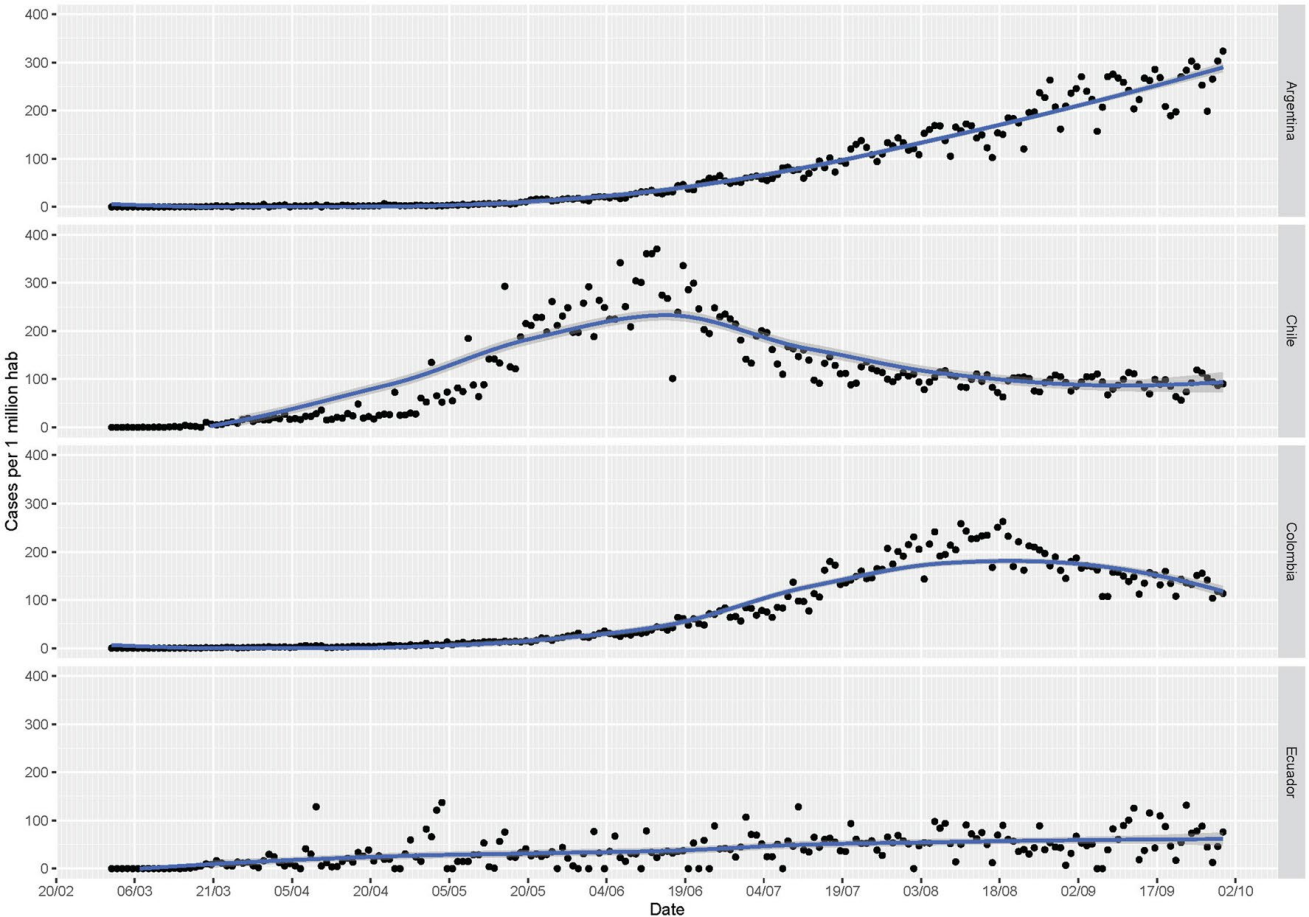
Confirmed cases rate in the four participating countries. Confirmed case rate per 1 million population from March 1 to September 30, 2020. The trend line is observed, and each point of dispersion corresponds to the daily record reported by each country. It can be seen how Argentina is climbing the curve of cases without reaching a peak. Chile had it between the dates of May 20 and June 30. Colombia from July 19 and currently, the cases seem to be decreasing and in Ecuador, which had its peak of cases in the month of April–May.

The incidence of daily confirmed cases per million inhabitants in each participating country has behaved differently, reaching its peak of cases at different times. As of September 30, 2020 (the cut-off date for the study), the curve was increasing in Argentina (rate case per 1 million inhabitants 249.7, SD 38.8), Chile reached its peak in May and June (206.1, SD 84.1), Colombia in July, and August (168, SD 54.8) and Ecuador in April (22.4, SD 24.4) (Fig. 1). The case fatality rate (September 30) for each country was 22.6; 27.5; 31.3; and 82.9 deaths per 1000 confirmed cases of SARS-CoV-2, respectively.

### Acute stress and the incidence of COVID-19

In Chile, Colombia, and Ecuador, the overall average score on the scale at the time of greatest emotional overload of the crisis (peak of daily cases) was higher than in the subsequent days when there was a decline in incidence (14.3 ± 5.3 vs. 6.9 ± 1.7, *p* < 0.05) (Table 2). For Argentina, the overall mean score on the scale corresponds to the time before the peak (8.7 CI95% 6.4–11) and the peak of cases (10.5 CI95% 9.5—11.5). Supplemental Table 2 includes detailed information on Argentina.

**Table 2:** Difference in global average at two-time points in the spread of the SARS-COV-2 pandemic in Chile, Colombia and Ecuador.

	Mean (95% CI)		*p*
	Peak incidence	Decreasing incidence	
Chile^a^ (N = 345)	14.3 (8.8–19.8)	6.9 (5.2–8.6)	0.07
Colombia^b^ (N = 365)	10.0 (8.7–11.3)	4.9 (3.6–6.2)	< 0.001
Ecuador^c^ (N = 209)	12.8 (11.4–14.2)	9.7 (8.3–11.1)	< 0.001

Mann–Whitney U test was performed for the comparison between groups (*p* < 0.05).

Scores from 0 to 30 in total on the scale.

Peak, highest incidence 7 days; Peak, lowest incidence 7 days.

^a^Peak: May 23 to Jun 30 2020; Peak: Jul 18 to Aug 20 2020.

^b^Peak: Jul 22 to Aug 18 2020; Peak: Aug 30 to Sep 30 2020.

^c^Peak: April 10 to May 15 2020; Decline: 4 to August 21 2020.

### Univariate analysis

Univariate analysis was conducted to identify possible cofounder variables of the outcomes of interest. As a result, women (*p* < 0.001), being younger than 50 years of age (*p* < 0.05) and COVID-19 infection and uncertainty of infection (*p* < 0.001) were identified as possible confounders (Table 3).

**Table 3:** Univariate analysis.

	Sex (Women)	Age (< 50 years)	COVID-19 infection (Yes/Do not know)
I can't help but think of recent critical situations. I can't get out of work	1.5 (1.4–1.6)^☩^	1.46 (1.4–1.5)*	1.56 (1.4–1.6)*
I have completely lost the taste for things that gave me peace of mind	1.17 (1.1–1.2)^☩^	1.12 (1.–1.2)*	1.27 (1.1–1.4)^☩^
I keep my distance, I resent dealing with people, I'm irascible even at home	1.04 (1–1.1)*	1.02 (0.9–1.1)*	1.11 (1–1.2)*
I feel that I am neglecting many people who need my help	0.92 (0.8–1)	0.92 (0.8–1)*	0.97 (0.9–1.1)*
I have difficulty thinking and making decisions, I have many doubts, I have entered a kind of emotional blockage	0.90 (0.8–1)^☩^	0.87 (0.8–0.9)*	0.99 (0.9–1.1)^☩^
I feel intense physiological reactions (shocks, sweating, dizziness, shortness of breath, insomnia, etc.) related to the current crisis	1.17 (1.1–1.2)^☩^	1.08 (1–1.2)*	1.27 (1.1–1.3)^☩^
I feel on permanent alert. I believe that my reactions now put other patients, my colleagues, or myself at risk	0.80 (0.7–0.9)	0.79 (0.7–0.9)*	0.84 (0.7–1)
Worrying about not getting sick causes me a strain that's hard to bear	1.20 (1.1–1.3)	1.17 (1.1–1.3)*	1.23 (1.1–1.4)
I'm afraid I'm going to infect my family	2.01 (1.9–2.1)*	2 (1.9–2.1)*	2.11 (2–2.2)^☩^
I have difficulty empathizing with patients' suffering or connecting with their situation (emotional distancing, emotional anaesthesia)	0.42 (0.3–0.5)	0.45 (0.4–0.5)*	0.50 (0.4–0.6)
Total score	11.13 (10.5–11.7)^☩^	10.88 (10.3–11.4)*	11.84 (11.1–12.6)^☩^
Factor 1. Affective response	5.95 (5.6–6.3)^☩^	5.84 (5.5–6.1)*	6.40 (5.9–6.9)^☩^
Factor 2. Fears and anxiety	5.18 (4.9–5.4)^☩^	5.04 (4.8–5.3)*	5.45 (5.1–5.8)^☩^

*Stands for *p* value < 0.05.

^☩^Stands for *p* value < 0.001.

Scores from 0 to 3 points on each of the items on the scale.

Scores from 0 to 30 in total on the scale.

Scores from 0 to 18 in factor 1.

Scores from 0 to 12 in factor 2.

### Acute stress and direct care of COVID-19 patients

Multivariable logistic regression was performed to analyze the correlations between the outcome of interest and possible cofounders. As a result, having less experience in the profession (*p* < 0.005) and working in a hospital environment (*p* < 0.001) were correlated with acute stress and care of COVID-19 patients. For this model, the sex of the healthcare worker did not have any interaction (Table 4).

**Table 4:** Multivariable logistic regression model for care of COVID-19 patients.

Variable	Exp (β)	Confidence interval	*p* value
Years in the profession (1 = less experience)	0.97	0.95–0.99	0.005
Sex (1 = women)	0.86	0.60–1.24	0.43
Place of work (1 = hospital)	2.42	1.51–3.87	0.001

Direct care in areas intended for COVID patients represented more distress for healthcare professionals. Specifically, participants worried about getting sick and the recent critical situations they had endured. As a result of the Kruskal–Wallis test, healthcare workers in COVID-19 critical care had higher scores than non-COVID-19 ward (*p* < 0.05). Table 5 shows the differences according to work area.

**Table 5:** Differences in the level of acute stress according to the work area.

	COVID-19 specific ward (N = 66)	COVID-19 Critical care (ICU and intermediate care) (N = 116)	Non-COVID-19 ward (N = 43)	*p*
I can't help but think of recent critical situations. I can't get out of work	1.5 (1.2–1.7)	1.8 (1.6–1.9)	1.3 (1.0–1.5)	0.040
I have completely lost the taste for things that gave me peace of mind	1.3 (1.0–1.5)	1.3 (1.1–1.5)	1.2 (0.9–1.5)	0.02
I keep my distance, I resent dealing with people, I'm irascible even at home	1.0 (0.8–1.2)	1.2 (1.0–1.4)	1.0 (0.7–1.3)	0.03
I feel that I am neglecting many people who need my help	1.0 (0.7–1.2)	1.0 (0.9–1.2)	1.0 (0.7–1.3)	0.02
I have difficulty thinking and making decisions, I have many doubts, I have entered a kind of emotional blockage	1.0 (0.7–1.2)	1.0 (0.9–1.2)	0.9 (0.7–1.2)	0.02
I feel intense physiological reactions (shocks, sweating, dizziness, shortness of breath, insomnia, etc.) related to the current crisis	1.3 (1.1–1.6)	1.5 (1.3–1.7)	1.1 (0.8–1.3)	0.01
I feel on permanent alert. I believe that my reactions now put other patients, my colleagues, or myself at risk	0.8 (0.6–1.1)	1.0 (0.8–1.2)	0.6 (0.4–0.9)	0.01
Worrying about not getting sick causes me a strain that's hard to bear	1.2 (1.0–1.4)	1.5 (1.3–1.7)	1.2 (0.9–1.5)	0.01
I'm afraid I'm going to infect my family	2.1 (1.8–2.3)	2.3 (2.1–2.4)	1.8 (1.6–2.1)	0.04
I have difficulty empathizing with patients' suffering or connecting with their situation (emotional distancing, emotional anaesthesia)	0.4 (0.2–0.5)	0.5 (0.3–0.6)	0.5 (0.3–0.7)	0.01
Total score	11.5 (9.9–13.0)	13.0 (11.8–14.2)	10.7 (8.8–12.6)	0.03
Factor 1. Affective response	6.0/18 (33.3%) (5.0–7.0)	6.8/18 (37.8%) (6.0–7.6)	6.9/18 (38.3%) (4.8–7.2)	0.07
Factor 2. Fears and anxiety	5.5/12 (45.8%) (4.8–6.2)	6.2/12 (51.7%) (5.7–6.7)	4.7/12 (39.2%) (3.8–5.6)	0.05

The Kruskal–Wallis test was performed for the comparison between groups (*p* < 0.05).

Scores from 0 to 3 points on each of the items on the scale.

Scores from 0 to 30 in total on the scale.

Scores from 0 to 18 in factor 1.

Scores from 0 to 12 in factor 2.

### Acute stress and SARS-CoV-2 infection

Multivariable logistic regression was performed to analyze the correlations between high emotional distress and acute stress (scores > 14) and possible cofounders. As a result, having less experience in the profession (*p* < 0.002), women healthcare workers (*p* < 0.05), and COVID-19 infection or uncertainty of being infected (*p* < 0.05) were correlated with acute stress. For this model, the place of work of the healthcare worker did not have any interaction (Table 6).

**Table 6:** Multivariable logistic regression model for acute stress and COVID-19 infection.

Variable	Exp (β)	Confidence interval	*p* value
Years in the profession (1 = less experience)	0.97	0.94–0.99	0.002
Sex (1 = women)	1.70	1.09–2.66	0.01
Place of work (1 = hospital)	1.40	0.82–2.36	0.22
COVID-19 infection (1 = yes/don’t know)	1.50	1.04–2.16	0.03

As a result of the Kruskal–Wallis test, acute stress levels were higher among professionals who were infected with SARS-CoV-2 compared to those who were not (Table 7). Furthermore, healthcare professionals that did not know if they had COVID-19 (uncertainty) were also higher when compared to professionals who were not infected. This result was consistent with the 10 items of the EASE Scale (*p* < 0.001).

**Table 7:** Differences in the level of acute stress depending on whether the professional was infected.

	No (N = 363)	Yes (N = 149)	Don't know (N = 126)	*p*
I can't help but think of recent critical situations. I can't get out of work	1.3 (1.2–1.4)	1.5 (1.4–1.6)	1.6 (1.4–1.8)	0.008
I have completely lost the taste for things that gave me peace of mind	0.9 (0.8–1.0)	1.3 (1.1–1.5)	1.3 (1.1–1.5)	0.001
I keep my distance, I resent dealing with people, I'm irascible even at home	0.9 (0.8–1.0)	1.0 (0.9–1.1)	1.2 (1.0–1.4)	0.004
I feel that I am neglecting many people who need my help	0.8 (0.7–0.9)	1.0 (0.9–1.1)	1.0 (0.8–1.2)	0.005
I have difficulty thinking and making decisions, I have many doubts, I have entered a kind of emotional blockage	0.7 (0.6–0.8)	1.0 (0.8– 1.2)	1.0 (0.8–1.2)	< 0.001
I feel intense physiological reactions (shocks, sweating, dizziness, shortness of breath, insomnia, etc.) related to the current crisis	0.9 (0.8–1.0)	1.4 (1.2–1.6)	1.2 (1.0–1.4)	0.03
I feel on permanent alert. I believe that my reactions now put other patients, my colleagues, or myself at risk	0.7 (0.6–0.8)	0.8 (0.7–0.9)	0.9 (0.7–1.1)	0.003
Worrying about not getting sick causes me a strain that's hard to bear	1.1 (1.0–1.2)	1.2 (1.0–1.4)	1.3 (1.1–1.5)	< 0.001
I'm afraid I'm going to infect my family	1.8 (1.7–1.9)	2.1 (1.9–2.3)	2.1 (1.9–2.3)	0.001
I have difficulty empathizing with patients' suffering or connecting with their situation (emotional distancing, emotional anaesthesia)	0.4 (0.3–0.5)	0.5 (0.4–0.6)	0.5 (0.4–0.6)	0.004
Total score	9.5 (8.8–10.2)	11.7 (10.7–12.7)	12.0 (10.8–13.2)	0.001
Factor 1. Affective response	5.0/18 (27.8%) (4.6–5.4)	6.3/18 (34.8%) (5.7–6.9)	6.6/18 (36.4%) (5–9–7.3)	0.05
Factor 2. Fears and anxiety	4.5/12 (37.7%) (4.2–4.8)	5.4/12 (45.2%) (4.9–5.9)	5.5/12 (45.7%) (5.0–6.0)	0.05

The Kruskal–Wallis test was performed for the comparison between groups (*p* < 0.05).

Scores from 0 to 3 points on each of the items on the scale.

Scores from 0 to 30 in total on the scale.

Scores from 0 to 18 in factor 1.

Scores from 0 to 12 in factor 2.

## Discussion

During the outbreak, one in three Latin American professionals reported they had experienced a medium to high level of acute stress, compatible with limited professional response capacity. These stress levels were higher at the worst moments of the outbreak regarding the incidence of new cases; and among professionals who believed they had been infected or were COVID-19 patients. The acute stress level was directly related to a major risk of infection due to caring in the front line COVID-19 patients. Indeed, workers in critical care units and COVID-19 wards showed higher scores in the EASE scales than others. Experience in healthcare setting has been identified as protective factor of acute stress. This confirms the impact of SARS-CoV-2 on healthcare professionals beyond the stress that healthcare may usually entail and reinforces the idea that healthcare professionals are the second most affected by the coronavirus. The results were consistent across countries.

As in other studies, the fear of infecting the family and not being able to disconnect from work once at home were the most severe sources of stress. If we compare the overall result of the scale in the Latin American countries with those obtained in Spain (11.1/30 points), the scores were very similar. In relation to studies that have used different scales, for instance, measuring burnout in professionals from 60 countries, they point out that acute stress reactions affected a third of the professionals while approximately 50% of the professionals in these countries manifested burnout symptoms^11^. These results add to those published by researchers from other countries, confirming the negative impact on the mental health of healthcare professionals from the COVID-19 pandemic^3,20,25,26^. This study highlights that although the pandemic has had different implications in countries and regions, the emotional response of healthcare professionals has been similar. It reinforces the idea that to deal with a pandemic; it is necessary to include measures and resources to provide emotional support to healthcare professionals to achieve an adequate response to the changing needs according to the evolution of the incidence required by patients (in this case, COVID-19 and not COVID-19). Training and emotional support to cope with the pandemic, appropriate protective measures, or clear information and protocols have been shown to mitigate this stress^5,16,19,27^.

EASE has been adapted to the linguistic and cultural context of Argentina, Colombia, Chile, and Ecuador. This tool combines reliability and construct validity suitable for screening acute stress reactions of healthcare professionals who care for COVID-19 patients who speak Latin American Spanish.

The scale includes a set of situations identified as the primary sources of stress and facilitates awareness of the impact of the pandemic on professionals, the second victims of SARS-CoV-2. Unlike other instruments that measure general anxiety or depression, the EASE focuses its content on distress in the care of COVID-19 patients. Furthermore, its length (10 elements) and the fact that it is linked to support mechanisms for professionals and teams, depending on the case, are provided through a web page and mobile app^13,16^, which prove to be other advantages. These data suggest that it can be used in the recovery phase of professionals and health systems to monitor professionals' responses after the impact of the pandemic. In this case, it can be expected that professionals' resilience will be more significant in the event of new outbreaks^28^. However, the reaction may differ depending on the support received during the first wave and the public response. Finally, if the health system is concerned about the welfare of its professionals^29^.

Through EASE, it has been possible to interpret that being in critical situations that do not allow them to disconnect from work and the fear of infecting your family when they get home are health professionals' main concerns and sources of stress. As reported in other studies, professionals in the direct care of COVID-19 patients showed higher emotional overload and distress levels^11^. Caring for the professional caregiver is a prerequisite for optimal care. The World Health Organization has identified the importance of the well-being of the healthcare providers and has announced new objectives for all healthcare systems in this direction. Studies like this reinforce this decision and show how the outbreak accelerates the need to implement measures that promote the welfare and work morale of the healthcare workforce for patients' benefit.

The levels of acute stress were also higher, coinciding with the moments of greatest incidence of COVID-19 cases, unlike what was observed in Spain. As predicted by the Community disaster response model^30^, acute stress was more significant during the restoration phase^4^. These differences could be because the pandemic's impact was not expected in Europe despite the data from Asian countries arriving, particularly China and Korea, in Latin American countries, it was intuited, and the lack of individual protection measures and fear of contagion was anticipated.

Monitoring stress seems advisable as interventions to strengthen the resilience of the health workforce have, so far, not achieved their goal. The reasons for this may vary, including resistance to participating in these techniques. Tools are needed to enable health professionals, especially men, to recognize the effect of the pandemic on their mental health. Almost 70% of the participants in this study were women, and this may be due to different reasons, including the fact that most health professionals are women, they tend to be more open to asking for help than men, and they participate more in this type of interventions. EASE can help monitor levels of acute stress and determine the degree of effectiveness of programmed interventions to reduce this stress, including those tested in other extreme situations^31^.

Limitations. The sample was not randomized, so a selection bias cannot be ruled out. Furthermore, convenience sampling was used, and participants were invited thru institutional mailings, instant messaging applications, and discussion forums. Individuals who chose to participate might systematically differ from those who did not, affecting generalizability. The survey used an online platform designed for this study or by downloading an application and was sent out to healthcare networks and hospitals in the participating countries; due to this, data on its reach is not known to establish an uptake percentage. There may be turns or grammatical expressions in Bolivia, Mexico, Peru, or other regional countries that are not covered by this adaptation. The availability of resources, mental health support, PPE provision, and the pandemic incidence between countries, territories of the same country, or between health centers can modify the responses and relationship of acute stress situations contemplated in the EASE scale. Approximately half of the respondents did not answer whether they had been infected. When interpreting these data, it is important to consider the diagnostic limitations that may exist in the region and how they may affect the rate of confirmed cases case fatality rate. If we compare the testing rate per million population in the United Kingdom (482,040) or the United States (505,045), the rate in Latin American countries is much lower (Chile 251,862; Colombia 89,101; Argentina 66,017; Ecuador 30,555)^24^.

In conclusion, this multinational study in Latin America shows that the infection affected healthcare workers' mental health. Twenty-seven percent of health care workers in Argentina, Colombia, Chile, and Ecuador experienced a medium to high level of acute stress following the outbreak. A higher intensity was observed among those working in COVID-19 critical care units and those who became infected or doubted whether they were infected with SARS-CoV-2. Acute stress increased as the incidence of COVID-19 cases increased. In future potential pandemics, this aspect should not surprise us, and from the very beginning, it is necessary to activate support measures to prevent this situation from negatively affecting patients.

## Supplementary Information

Supplementary Information.

## Data Availability

Data are available upon reasonable request.
