# Author Correction: Contributing factors for acute stress in healthcare workers caring for COVID-19 patients in Argentina, Chile, Colombia, and Ecuador

**DOI:** 10.1038/s41598-022-13900-z

**Published:** 2022-06-07

**Authors:** Jimmy Martin-Delgado, Rodrigo Poblete, Piedad Serpa, Aurora Mula, Irene Carrillo, Cesar Fernández, María Asunción Vicente Ripoll, Cecilia Loudet, Facundo Jorro, Ezequiel Garcia Elorrio, Mercedes Guilabert, José Joaquín Mira

**Affiliations:** 1 Atenea Research Group, Foundation for the Promotion of Health and Biomedical Research, Hospital Universitario de Sant Joan d’Alacant, Carretera Nacional 332, 03550 Sant Joan d’Alacant, Alicante, Spain; 2 Instituto de Investigación E Innovación en Salud Integral, Facultad de Ciencias Médicas, Universidad Católica de Santiago de Guayaquil, Guayaquil, Ecuador; 3 Pontifical Catholic University of Santiago de Chile, Región Metropolitana, Chile; 4 Universidad de Santander, Bucaramanga, Colombia; 5 Department of Health Psychology, Miguel Hernández University, Elche, Spain; 6 General José de San Martín de La Plata General Hospital, Buenos Aires, Argentina; 7 Institute for Clinical Effectiveness and Health Policy (IECS), Buenos Aires, Argentina; 8 Hospital General de Niños Pedro de Elizalde, Buenos Aires, Argentina; 9 Health District Alicante-Sant Joan, Alicante, Spain

Correction to: *Scientific Reports* https://doi.org/10.1038/s41598-022-12626-2, published online 19 May 2022

The Funding section in the original version of this Article was incomplete.

"This study was supported by PROMETEO/2021/061 grant (Generalitat Valenciana, 2021). J.M.D., R.P., P.S., I.C., E.G.E., M.G., and J.J.M. were supported by The European Cooperation in Science and Technology, COST [Grant Number CA19113]."

now reads:

"This study was supported by Generalitat Valenciana, 2021. CONV. UMH-GVA REF. SOLCIF 2020/0005. (Cód. Sub. 11-134-4-2021-0068). J.M.D., R.P., P.S., I.C., E.G.E., M.G., and J.J.M. were supported by The European Cooperation in Science and Technology, COST [Grant Number CA19113]."

The original Article has been corrected.

