# Supplementary material for: Contributing factors for acute stress in healthcare workers caring for COVID-19 patients in Argentina, Chile, Colombia, and Ecuador
Source: Sci Rep. 2022 May 19;12:8496. doi: 10.1038/s41598-022-12626-2 (PMC9119382; doi:10.1038/s41598-022-12626-2)
Supplement: Supplementary file 1 — Supplementary Information. [file 41598_2022_12626_MOESM1_ESM.docx]

**Supplemental table 1. Answers in each of the EASE items**

|  | **Mean (95% CI)** | **SD** | **It often happens to me (%)** | **I am like this all the time (%)** |
| --- | --- | --- | --- | --- |
| I can't help but think of recent critical situations. I can't get out of work. | 1.4 (1.3 – 1.5) | 1.0 | 26.4 | 15.2 |
| I have completely lost the taste for things that gave me peace of mind. | 1.0 (0.9 – 1.1) | 1.0 | 23.4 | 7.7 |
| I keep my distance, I resent dealing with people, I'm irascible even at home. | 1.0 (1.0 – 1.0) | 0.9 | 18.7 | 8.7 |
| I feel that I am neglecting many people who need my help. | 0.9 (0.9 – 1.1) | 1.0 | 20.5 | 7.1 |
| I have difficulty thinking and making decisions, I have many doubts, I have entered a kind of emotional blockage. | 0.8 (0.7 – 0.9) | 1.0 | 14.9 | 7.9 |
| I feel intense physiological reactions (shocks, sweating, dizziness, shortness of breath, insomnia) related to the current crisis. | 1.0 (0.9 – 1.1) | 1.0 | 20.8 | 11.3 |
| I feel on permanent alert. I believe that my reactions now put other patients, my colleagues, or myself at risk. | 0.9 (0.9 – 1.1) | 1.0 | 15.9 | 10.5 |
| Worrying about not getting sick causes me a strain that is hard to bear. | 1.1 (1.0 – 1.2) | 1.0 | 17.5 | 13.3 |
| I am afraid I'm going to infect my family. | 1.8 (1.7 – 1.9) | 1.1 | 25.7 | 35.3 |
| I have difficulty empathising with patients' suffering or connecting with their situation (emotional distancing, emotional anaesthesia). | 0.6 (0.6 – 0.6) | 0.9 | 10.3 | 6.0 |
| Total score | 10.6 (10.2 – 11.0) | 6.9 |  |  |
| Factor 1. Affective response | 5.7/18 (31.8% of total score) (5.5 - 5.9) | 4.2 |  |  |
| Factor 2. Fears and anxiety | 4.8/12 (40.3% of total score) (4.6 - 5.0) | 3.3 |  |  |

N=1372

Scores from 0 to 3 points on each of the items on the scale

Scores from 0 to 30 in total on the scale

Scores from 0 to 18 in factor 1

Scores from 0 to 12 in factor 2

**Supplemental table 2. Difference in global average at two-time points in the spread of the SARS-COV-2 pandemic in Argentina**

|  | Average (95% CI) | | |  |
| --- | --- | --- | --- | --- |
|  | Previous start of incidence | | Peak incidence | p |
| Argentina (N=375) | 8.7 (6.4 – 11.0) | 10.5 (9.5 – 11.5) | | 0.3 |

Mann-Whitney U test was performed for the comparison between groups (p<0.05)

Scores from 0 to 30 in total on the scale

Previous less incidence 7 days; Peak more incidence 7 days

^d^ Preview: May 20 to Jul 2 2020; peak: 1 to Sep 21 2020
